# Economic Burden for Lung Cancer Survivors in Urban China

**DOI:** 10.3390/ijerph14030308

**Published:** 2017-03-15

**Authors:** Xin Zhang, Shuai Liu, Yang Liu, Jian Du, Wenqi Fu, Xiaowen Zhao, Weidong Huang, Xianming Zhao, Guoxiang Liu, Zhengzhong Mao, Teh-wei Hu

**Affiliations:** 1 School of Public Health, Harbin Medical University, 157 Baojian Road, Nangang District, Harbin 150086, China; zhangxinzhx0801@126.com (X.Z.); liuyangly1989@163.com (Y.L.); fuwenqi6688@163.com (W.F.); mary0822@163.com (X.Z.); weidong218@126.com (W.H.); 2 Graduate School of China Academy of Chinese Medical Sciences, Beijing 100700, China; smile_shuaishuai@hotmail.com; 3 Department of Clinic Medicine, Heilongjiang Nursing College, 209 Xuefu Road, Nangang District, Harbin 150086, China; dujian.0317@163.com; 4 Chinese People’s Liberation Army 211 hospital, Harbin 150080, China; amingk@163.com; 5 West China School of Public Health, Sichuan University, Chengdu 610041, China; zzmao666@yahoo.com; 6 School of Public Health, University of California, Berkeley 94720, CA, USA; thu@berkeley.edu

**Keywords:** economic burden, lung cancer, direct cost, indirect cost, medical insurance, China

## Abstract

*Background*: With the rapid increase in the incidence and mortality of lung cancer, a growing number of lung cancer patients and their families are faced with a tremendous economic burden because of the high cost of treatment in China. This study was conducted to estimate the economic burden and patient responsibility of lung cancer patients and the impact of this burden on family income. *Methods*: This study uses data from a retrospective questionnaire survey conducted in 10 communities in urban China and includes 195 surviving lung cancer patients diagnosed over the previous five years. The calculation of direct economic burden included both direct medical and direct nonmedical costs. Indirect costs were calculated using the human capital approach, which measures the productivity lost for both patients and family caregivers. The price index was applied for the cost calculation. *Results*: The average economic burden from lung cancer was $43,336 per patient, of which the direct cost per capita was $42,540 (98.16%) and the indirect cost per capita was $795 (1.84%). Of the total direct medical costs, 35.66% was paid by the insurer and 9.84% was not covered by insurance. The economic burden for diagnosed lung cancer patients in the first year following diagnosis was $30,277 per capita, which accounted for 171% of the household annual income, a percentage that fell to 107% after subtracting the compensation from medical insurance. *Conclusions*: The economic burden for lung cancer patients is substantial in the urban areas of China, and an effective control strategy to lower the cost is urgently needed.

## 1. Introduction

Lung cancer has been a major public health problem in most countries for several decades. In 2012, an estimated 1.8 million new cases of lung cancer were diagnosed (12.9% of the total new cancer cases); 58% of these cases occurred in the less developed regions of the world. Lung cancer was the most common cause of death from cancer worldwide, responsible for an estimated nearly one in five cancer deaths (1.59 million deaths, 19.4% of the total cancer deaths) [1].

In China, lung cancer has been the leading cancer diagnosis and the leading cause of cancer deaths for many years. According to the Chinese National Central Cancer Registry (NCCR), the year 2010 saw 605,946 new lung cancer diagnoses (19.59% of the total cancer cases) in China with a crude incidence rate of 46.08 per 100,000. Of these diagnoses, the number of cases from urban areas was 348,107 (57.45% of the total new lung cancer cases). With respect to mortality from cancer, an estimated 486,555 people in China died of lung cancer in 2010 with a crude mortality rate of 37.00 per 100,000, and 279,919 (57.53%) of these patients were from urban areas [2]. The World Health Organization (WHO) predicted that by 2025, the annual number of new cases of lung cancer mortality in China will be over one million, and the number of lung cancer patients will be the highest in the world [3].

As the population ages, the expenditure for lung cancer treatment will become even more burdensome to the entire society [4,5,6]. To date, a relatively large amount of research has been done on the economic burden of lung cancer worldwide [7,8,9,10,11,12,13,14,15,16]. However, the economic burden of lung cancer patients varies by country, due to differences in economic development, health systems, exchange rate, purchasing power, and other factors [5].

Studying the economic burden of lung cancer in China is attracting the attention of more and more scholars. However, most studies to date have been limited in measuring the direct costs or hospitalization expenses of lung cancer patients by using data from specific hospitals [17,18,19,20,21]. Moreover, most previous studies also were limited in calculating short-term costs without considering the cost of long-term treatment in the years following diagnosis.

According to previous reports, the five-year survival rate of lung cancer patients in China is reported at 10%–15% [3,18,22]. Estimating the expenditure of lung cancer patients in the first five years is necessary to grasp the burden of the whole course of lung cancer treatment. Therefore, the aim of this study is to calculate the total cost of lung cancer treatment for lung cancer survivors in China within five years from the date of diagnosis. The total cost includes direct costs and indirect costs. In addition, we analyzed the payment of health insurance schemes on the direct medical costs and the economic impact of lung cancer on the family income, information that could provide an objective basis for health care policymaking.

## 2. Methods

### 2.1. Data Source

In this study, the sample was identified through the Nangang District Registry information system of the Chinese National Central Cancer Registry [23]. From 2010 to 2014, 396 individuals survived a diagnosis of lung cancer in 10 communities of Nangang District. Among them, 195 lung cancer patients were finally enrolled in the study; the remainders were removed from the potential sample because of the researchers’ inability to get follow-up data and/or patients’ refusal to participate. The retrospective questionnaire survey was conducted with 195 surviving lung cancer patients or with their main family caregivers to collect the information on sociodemographic characteristics, utilization of medical services, and costs during illness. All of the respondents provided informed consent in writing.

### 2.2. Study Patient Identification

Cases included in the study were surviving patients who had been diagnosed with lung cancer defined according to the International Classification of Disease 10th revision (ICD-10) codes from C33-C34 [24].

### 2.3. Cost Classification and Definition

In this study, the economic burden of lung cancer was estimated by calculating direct medical costs, direct nonmedical costs, and indirect costs covered in the five years following diagnosis. Direct medical costs were expenditures on medical services and drugs associated with diagnosis and treatment performed in hospitals, clinics, and pharmacies. These direct costs included three components: inpatient cost, outpatient cost, and purchased drug cost for lung cancer treatment paid by insurance, co-payment, and non-insurance [25]. Direct nonmedical costs included transportation costs for a round trip to visit health service providers and costs associated with accommodations, extra nutrition, and hired escorts for lung cancer patients during the period of treatment in medical institutions. Indirect costs were defined as days of lost productivity for both patients and their family caregivers resulting from outpatient visits and hospitalization due to lung cancer [16].

### 2.4. Cost Estimation

The direct cost was calculated by summing the cost of the episodes of care in the period considered in this study. Because the costs of treatments varied in different years, all the costs were adjusted to 2014 to eliminate the effects of inflation using the annual consumer price index (CPI) of Heilongjiang Province [26,27].

Indirect costs were measured using the human capital approach. The indirect costs were calculated by multiplying the average daily wage income for an urban resident in Heilongjiang Province from 2010 to 2014 [16,28,29]. In China, the mandatory retirement age for men is 60, and, for women, it is 50. Therefore, the productivity loss for patients and their families beyond this age range were not considered in this study. Since the lost wages were considered over five years, a 3% inflation index was used to convert past monetary values into present value in 2014 [6,30]. The Medical Ethics Committee of Harbin Medical University (Daqing) agreed with this study and examined the project for related medical ethics problems. The ethical project identification code is 16HMUSCI032.

## 3. Results

### 3.1. Description of Patient Sociodemographic Characteristics

As noted, 195 patients with lung cancer were eventually included in this study. Study participants at the time of the survey were aged 29 to 89; 122 (62.56%) of them were male; 125 (64.10%) had retired, 22 (11.28%) were employed, and about a quarter were unemployed. No statistical significance was found at the 5% level between respondents and identified patients not included in the sample by age (*p* = 0.898) and gender (*p* = 0.311). Of the 195 patients included in the study, 157 respondents (80.51%) were covered by various health insurance schemes and 38 (19.49%) were uninsured (Table 1).

### 3.2. Economic Burden of Lung Cancer

#### 3.2.1. Direct Medical Costs

The direct medical costs to the lung cancer patients in the study within five years after diagnosis were $40,650 per patient, making up 93.80% of the total costs. The average total cost of outpatient visits for those who made such visits was $1679 per patient, accounting for 3.87% of the average total costs. In addition, the direct medical cost for hospitalization was $28,307 per lung cancer patient, representing 65.32% of the total costs of treatment. The cost of drugs associated with lung cancer treatment that were purchased from a pharmacy was $10,664 per patient, comprising 24.61% of the total costs. In summary, hospitalization was the main component of the direct medical costs for lung cancer patients, followed by the cost of purchased drugs (Table 2).

#### 3.2.2. Direct Nonmedical Costs

The average direct nonmedical cost was $1890 per patient, which accounted for 4.36% of total costs (Table 2). The largest direct nonmedical cost was the nutrition costs, $1300 per patient, higher than the accommodation costs and transportation costs.

#### 3.2.3. Indirect Costs

The average indirect cost calculated as the lost productivity of patients and family caregivers was $795 and represented the smallest share of overall costs at 1.84%. The average cost of lost productivity was considerably higher for family caregivers ($704) than for the patients themselves ($92) (Table 2).

### 3.3. Influence of Medical Insurance on the Economic Burden of Lung Cancer Patients

A total of $7,926,775 was spent as direct medical costs for the 195 patients in the sample, $780,085 (9.84%) of which was paid by 38 noninsured patients. The direct medical costs for patients covered by medical insurance were paid jointly by insurance and as copayments by insured patients. Of the total direct medical expenditures for lung cancer, medical insurance paid for 22.98% of the outpatient costs, 48.86% of the hospitalization costs, and 2.59% of the costs of purchased drugs, respectively (Table 3).

Of the 195 lung cancer patients in the study, 157 participated in medical insurance schemes, and the average reimbursement rate of direct medical costs for insured patients was 39.55%. The highest reimbursement rate for direct medical costs was other types of insurance, which included commercial medical insurance, two or more forms of medical insurance, and so forth. Of the 195 participants, 141 patients were covered by Urban Employees Basic Medical Insurance (UEBMI), with a reimbursement rate of 39.39%, followed by the reimbursement rate of 38.14% from Urban Residents Basic Medical Insurance (URBMI) (Table 4).

### 3.4. Impact of Economic Burden on the Family Economic Situation

The proportion of the economic burden of lung cancer treatment to household income during the first year after diagnosis decreased from 171% to 107% after health insurance reimbursement. The impact of the economic burden on the family economic situation varied with the quintile household income. The results showed that the higher the household income, the lower the economic burden of lung cancer relative to household income. For the low- and middle-income households, the economic burden of the first year’s treatment was still higher than the annual household income after the reimbursement of medical insurance (Table 5).

## 4. Discussion

This paper was designed to estimate the economic burden of lung cancer treatment within five years from the date of diagnosis for lung cancer survivors in urban China. The total economic burden was $43,336 per patient and was concentrated mainly in the first year after diagnosis. The study showed that direct medical costs were the major component of the total economic burden of patients with lung cancer. The average direct medical cost in the first year after diagnosis was $30,277. Prior studies that estimated the direct economic burden of lung cancer inpatients showed that the average direct cost ranged from $16,276 to $20,076 during the period of hospitalization in other areas of China [32,33,34]. These estimates are considerably lower than those identified in our study because their direct medical costs were collected from medical records in specific hospitals only. Of the direct medical costs, the proportion of hospitalization costs to the total economic burden of lung cancer was higher than other costs. The average hospitalization cost that we estimated was $28,307, accounting for 65.32% of the total economic burden, followed by purchased drugs, which accounted for 24.61%. The study by Vasiliki et al. indicated that hospitalization costs ranged from 31% to 71% of total costs based on findings from several other countries [16]. The results of our study suggest that more attention should be paid to the management of hospitalization costs and to the cost of drugs from pharmacies for lung cancer patients.

The average indirect cost found in this study was $795, accounting for 1.84% of the total economic burden. This figure is probably an underestimate for several reasons. First, lost working days were considered a time loss only during the treatment period; the days lost by patients and their families during the recovery period were not included in our study due to lack of reliable data; Second, productivity losses for males older than 60 and for females over 50, the official retirement ages in China, were not calculated in the indirect cost. The relatively high ages of lung cancer patients might account for the small indirect costs. Third, we estimated productivity lost by using the unified wage standard without considering the different level of wages in different professions.

Although some costs are paid by medical insurance, the economic burden of the first year still accounted for 107.49% of the average family annual income after the insurance compensation. All of the families suffered catastrophic health expenditures, defined as out-of-pocket spending for health care that exceeds 40% of a household’s income [35,36]. A study by Park et al. reported that the cost for five-year lung cancer survivors constituted 44.7% of the per capita income during the same period in a tertiary care hospital in South Korea in 2002 [15]. The results of our study showed that the economic burden of lung cancer remains heavy, especially for low-income families in urban China. Thus, the government needs to strengthen the Fiscal Medical Assistance for these lung cancer patients.

Medical insurance played an important role in reducing the proportion of out-of-pocket expenses in direct medical costs. In our study, more than one-third of the direct medical costs were paid by medical insurance, and the costs paid by copayments of insured patients and noninsured patents were 54.5% and 9.84%, respectively. In China, urban residents are covered mainly by two social basic medical insurance systems: UEBMI, which covers employed urban residents, and URBMI, which is designed to cover nonemployed urban residents, children, and students [37]. Combined, UEMBI and URBMI covered about 71.35% of the urban population, with coverage rates of 39.30% and 32.04%, respectively, in Heilongjiang Province in 2014 [38]. The results of this study showed slightly different reimbursement ratios for lung cancer patients: 39.39% for UEBMI and 38.14% for URBMI patients. Of the 157 patients with health insurance, the reimbursement rate of health insurance was 39.55%, and the remaining 60.45% of direct medical costs were paid Out-of-Pocket. The high copayment for current health insurance schemes needs to be reduced to avoid the occurrence of catastrophic health expenditures.

This analysis has several limitations. First, the recall bias of respondents in reviewing the costs could not be avoided due to the retrospective investigation in the community; Second, because this study included only survivors of lung cancer, the indirect costs consisted of only the productivity loss caused by delays, and not the cost of lost productivity due to premature mortality, which could greatly undervalue the indirect economic burden of illness [39,40]. The lack of data on productivity lost by patients and caregivers during the recovery period could also underestimate the indirect cost estimates; Third, intangible economic costs to the psychological and mental health of lung cancer patients and their caregivers, such as the pain, sorrow, and inconvenience due to the decline in quality of life, were not included because they are difficult to convert into a monetary value [41]; Finally, the variety of clinical types and stages of lung cancer were not taken into account because of a lack of data, but they comprise a topic worthy of further studies.

## 5. Conclusions

From the perspective of the survival of individuals with lung cancer, this study showed that the costs of lung cancer are substantial compared to the patients’ household income. The economic burden of lung cancer patients is attributed mainly to direct costs in the urban areas of China. Furthermore, the largest component of the total economic burden was the cost of hospitalization. Indirect costs were considerably higher for family caregivers than for patients themselves and represented a relatively small proportion of the total economic burden. Although medical insurance paid on average 40% of the total direct medical cost for insured patients, the proportion of out-of-pocket expenses was still too high. The economic burden for lung cancer patients was heaviest in the first year following diagnosis and was likely to induce catastrophic health expenditures for households, especially those low- and middle-income families. To further reduce the health care economic burden, increased and well-targeted subsidies for low-income patients are needed.

## Figures and Tables

**Table 1:** Sociodemographic characteristics of 195 lung cancer patients.

Sociodemographic Characteristics	N	Male (n = 122)		Female (n = 73)	
		Number	Percent (%)/Mean	Number	Percent (%)/Mean
Age (years)					
0–39	2	0	0.00	2	2.74
40–49	11	8	6.56	3	4.11
50–59	53	32	26.23	21	28.77
60–69	78	56	45.90	22	30.14
≥70	51	26	21.31	25	34.25
Marital status					
Spinsterhood	1	1	0.82	0	0.00
Married/cohabitation	162	109	89.34	53	72.60
Divorce/separation	7	5	4.10	2	2.74
Widowed	25	7	5.74	18	24.66
Education status					
Primary school or below	27	12	9.84	15	20.55
Junior school	80	56	45.90	24	32.88
Senior school/technical	63	35	28.69	28	38.36
College degree or above	25	19	15.57	6	8.22
Employment status					
Employed	22	15	12.30	7	9.59
Unemployed	48	33	27.05	15	20.55
Retirement	125	74	60.66	51	69.86
Quintile household per capita income (2014) ($)					
Lowest	39	23	3541.72	16	4045.77
Low	39	26	5828.00	13	5633.55
Middle	39	24	6947.56	15	6941.10
Higher	39	23	9064.98	16	8821.72
Highest	39	26	14,637.02	13	14,354.52
Medical insurance					
Yes	157	97	79.51	60	82.19
No	38	25	20.49	13	17.81

Purchasing power parity (PPP) was used in this study to convert RMB to dollars: $1 = 3.567 RMB (The PPP values of RMB against the dollar in 2014) [31].

**Table 2:** Total economic burden of 195 lung cancer patients.

Type of Costs	Costs ($)	Cost per Patient ($)	95% Confidence Interval		%
			Lower Limit	Upper Limit	
Direct medical costs					
Outpatient	327,379.57	1678.87	1269.74	2088.00	3.87
Inpatient	5,519,906.27	28,307.21	24,337.95	32,276.48	65.32
Purchased drugs *	2,079,489.49	10,664.05	7521.11	13,806.99	24.61
Total	7,926,775.32	40,650.13	34,814.63	46,485.63	93.80
Direct nonmedical costs					
Transportation	10,809.01	55.43	28.88	81.98	0.13
Accommodation	36,290.37	186.10	121.29	250.92	0.43
Nutrition	253,551.65	1300.26	1021.32	1579.21	3.00
Caregiver	6674.45	34.23	1.44	67.02	0.08
Other **	61,269.82	314.21	200.46	427.95	0.73
Total	368,595.30	1890.23	1510.20	2270.27	4.36
Indirect costs					
Patient	17,844.32	91.51	63.84	119.18	0.21
Family caregivers	137,268.71	703.94	614.12	793.77	1.62
Total	155,113.03	795.45	692.97	897.93	1.84
Total economic burden	8,450,483.65	43,335.81	37,329.58	49,342.05	100.00

* The cost of purchased drugs was the expense of the drugs that patients bought in pharmacies for their own lung cancer treatment after the initial diagnosis. Expenditures for medicine prescribed by the doctors and purchased in medical institutions were included in outpatient or inpatient costs; ** The other costs were estimated including the cost of obtaining copies of medical records, the cost of special diets, special clothes, and wheelchairs for patients, etc. $1 = 3.567 RMB (the PPP values of RMB against the dollar in 2014).

**Table 3:** Medical expenditures payment by type of medical service.

Type of Service	Per capita Direct Medical Costs ($) (n = 195)	Paid by Insurer (n = 157)		Paid by Patients ** (n = 157)		Non-Covered *** (n = 38)	
		Per Capita Costs ($)	Percent * (%)	Per capita Costs ($)	Percent * (%)	Per capita Costs ($)	Percent * (%)
Outpatient	1678.87	479.24	22.98	1246.21	59.76	1486.42	17.25
Inpatient	28,307.21	17,179.75	48.86	14,196.67	40.38	15,626.5	10.76
Purchased drugs	10,664.05	343.41	2.59	12,075.04	91.17	3415.59	6.24
Total	40,650.13	18,002.40	35.66	27,517.92	54.50	20,528.55	9.84

Unit: USD; * Total cost paid by insurer or patients as a percent of total direct medical costs; ** Paid by the copayment of insured patients; *** Non-covered refers to the costs for services paid by patients without any medical insurance.

**Table 4:** Direct medical costs paid by type of medical insurance for insured patients.

Insurance Systems	n	Per Capita Direct Medical Costs ($)	Per Capita Cost Paid by Insurance ($)	Reimbursement Rate (%)
UEBMI	141	47,796.94	18,828.02	39.39
URBMI	8	28,685.56	10,941.92	38.14
Other types	8	22,229.71	10,511.34	47.29
Total	157	45,520.32	18,002.40	39.55

Unit: USD.

**Table 5:** Influence of the financial burden in the first year after diagnosis on the family economic situation.

Quintile Household Income	Average Household Income for 2014 (USD/Household) (1)	Average Economic Burden (USD/Patient) (2)	Average Reimbursement (USD/Patient) (3)	The Proportion of the Economic Burden of Household Income (%)	
				Before Reimbursement (4)	After Reimbursement (5)
Lowest	8083.07	19,073.03	4901.90	235.96	175.32
Low	12,854.30	30,650.86	11,855.12	238.45	146.22
Middle	16,684.28	34,316.62	14,103.15	205.68	121.15
Higher	20,870.80	26,960.39	10,390.47	129.18	79.39
Highest	29,882.90	40,382.12	15,138.53	135.13	84.48
Total	17,675.07	30,276.60	11,277.83	171.30	107.49

Note: Column 4 = column 2/column 1; column 5 = (column 2 − column 3)/column 1.
